# From primordial germ cells to primordial follicles: a review and visual representation of early ovarian development in mice

**DOI:** 10.1186/s13048-016-0246-7

**Published:** 2016-06-21

**Authors:** Hannah M. Wear, Matthew J. McPike, Karen H. Watanabe

**Affiliations:** Institute of Environmental Health, Oregon Health & Science University, 3181 SW Sam Jackson Park Rd. Mail code HRC3, Portland, OR 97239 USA; School of Public Health, Oregon Health & Science University, 3181 SW Sam Jackson Park Rd. Mail code GH230, Portland, OR 97239 USA

**Keywords:** Early ovarian development, Conceptual model, Mouse, Molecular signaling

## Abstract

**Background:**

Normal development of reproductive organs is crucial for successful reproduction. In mice the early ovarian developmental process occurs during the embryonic and postnatal period and is regulated through a series of molecular signaling events. Early ovarian development in mice is a seventeen-day process that begins with the rise of six primordial germ cells on embryonic day five (E5) and ends with the formation of primordial follicles on postnatal day two (P2).

**Results:**

We reviewed the current literature and created a visual representation of early ovarian development that depicts the important molecular events and associated phenotypic outcomes based on primary data. The visual representation shows the timeline of key signaling interactions and regulation of protein expression in different cells involved in ovarian development. The major developmental events were divided into five phases: 1) origin of germ cells and maintenance of pluripotency; 2) primordial germ cell migration; 3) sex differentiation; 4) formation of germ cell nests; and 5) germ cell nest breakdown and primordial follicle formation.

**Conclusions:**

This review and visual representation provide a summary of the current scientific understanding of the key regulation and signaling during ovarian development and highlights areas needing further study. The visual representation can be used as an educational resource to link molecular events with phenotypic outcomes; serves as a tool to generate new hypotheses and predictions of adverse reproductive outcomes due to perturbations at the molecular and cellular levels; and provides a comprehendible foundation for computational model development and hypothesis testing.

## Introduction

Successful reproduction in males and females is vital to survival and is dependent on several factors including development of reproductive organs. For females, normal development of the ovaries includes multiple cell types (e.g., oogonia, theca and granulosa cells) that provide structure and function vital for reproduction. Ovarian development in mammals can be split into two phases, 1) growth of the ovary and establishment of the founding primordial follicle pool and 2) maturation of follicles and release of a fertilizable egg starting at puberty. The first phase of ovarian development, or early ovarian development, occurs predominantly during the embryonic period. Explicitly, early ovarian development in mice begins on embryonic day five (E5) and finishes on postnatal day two (P2). Development of the ovaries is a highly regulated process dependent on a series of molecular signaling factors. Disruption in the signaling can lead to adverse reproductive outcomes, such as a smaller primordial follicle pool, incomplete development of follicles, and failure of normal sexual differentiation [1, 2]. Recognizing the importance of molecular signaling and the associated phenotypic outcomes is vital for understanding ovarian development.

Visual representations can be helpful for understanding complex concepts like molecular signaling networks. We created a visual representation of early ovarian development to highlight key molecular signaling processes and corresponding phenotypic outcomes. Many factors are involved in development of the gonad, however we chose to only include signaling events that are critical to producing viable oocytes and that are susceptible targets for reproductive toxicity. Starting on embryonic day five (E5) and ending on postnatal day two (P2), molecular events and phenotypic outcomes that occur on a given day within corresponding cell types are shown in the figures. All materials used to develop the figures were based on data from primary literature. Studies were chosen based on quality of research and species studied. In our review, early ovarian development in mice was divided into five different stages: 1) origin of germ cells and maintenance of pluripotency; 2) germ cell migration; 3) sex differentiation; 4) formation of germ cell nests; and 5) germ cell nest breakdown and primordial follicle formation.

A few days after birth, neo-oogenesis and formation of primordial follicles were traditionally believed to permanently cease, meaning the germ cell pool size in mammalian females is fixed at birth. Emerging studies have shown evidence for the presence of active germ stem cell types, very small embryonic-like stem cells (VSELs), female germline stem cells (FGSCs), ovarian stem cells (OSCs), ovarian germ stem cells (OGSCs), etc., in the postnatal ovary in several mammalian species, with the potential to contribute to the germ cell pool [3–6]. VSELs have been observed in studies with similar characteristics to developing primordial germ cells (e.g. maintenance of pluripotency) giving rise to progenitors that are associated with increased meiosis and appearance of primordial follicles in the adult mouse ovary [7, 8], however other studies continue to claim absence of active germ cells in the postnatal ovary [9–11]. Though the controversy over the role of OSCs is important in understanding the size of the germ cell pool and female reproduction, it is outside the scope and timeline of our focus on early ovarian development in mice.

## Stages of early ovarian development and visual representation

### Origin of germ cells and maintenance of pluripotency factors (E5 to E7)

**Fig. 1:**
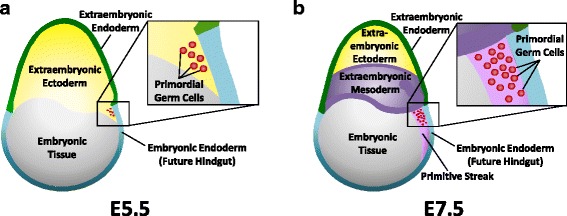
Embryonic tissues in early development. **a** First appearance of six primordial germ cells (red) in the posterior extraembryonic ectoderm (*yellow*) near the embryonic endoderm (*blue*) of a mouse embryo (*gray*) on embryonic day 5.5 (E5.5). **b** On E7.5, the population of primordial germ cells has increased and are present in the primitive streak (*magenta*) near the extraembryonic mesoderm (*purple*) and embryonic endoderm (*blue*) of the mouse embryo (*gray*)

Primordial germ cells (PGCs) are diploid cells that give rise to the germline in both males and females [2, 12, 13]. They are the precursors of oocytes and spermatozoa in the ovaries and testes, respectively. Six PGCs arise on E5 from a cell population located in the extraembryonic ectoderm (Fig. 1a). PGCs undergo mitosis, dividing into a larger population of undifferentiated cells. By E7, about 40 PGCs are detected in primitive streak in the cleft between the yolk sac and developing allantois near posterior the extraembryonic mesoderm tissue (Fig. 1b) [14]. Extraembryonic tissues in mammals are important for fetal development, though they do not remain attached after birth. PGCs are the only cell type to originate outside of embryonic tissue that remains in the animal after birth. It is hypothesized that PGCs are located external to embryonic tissue to avoid differentiation signals and maintain pluripotency [15].

**Fig. 2:**
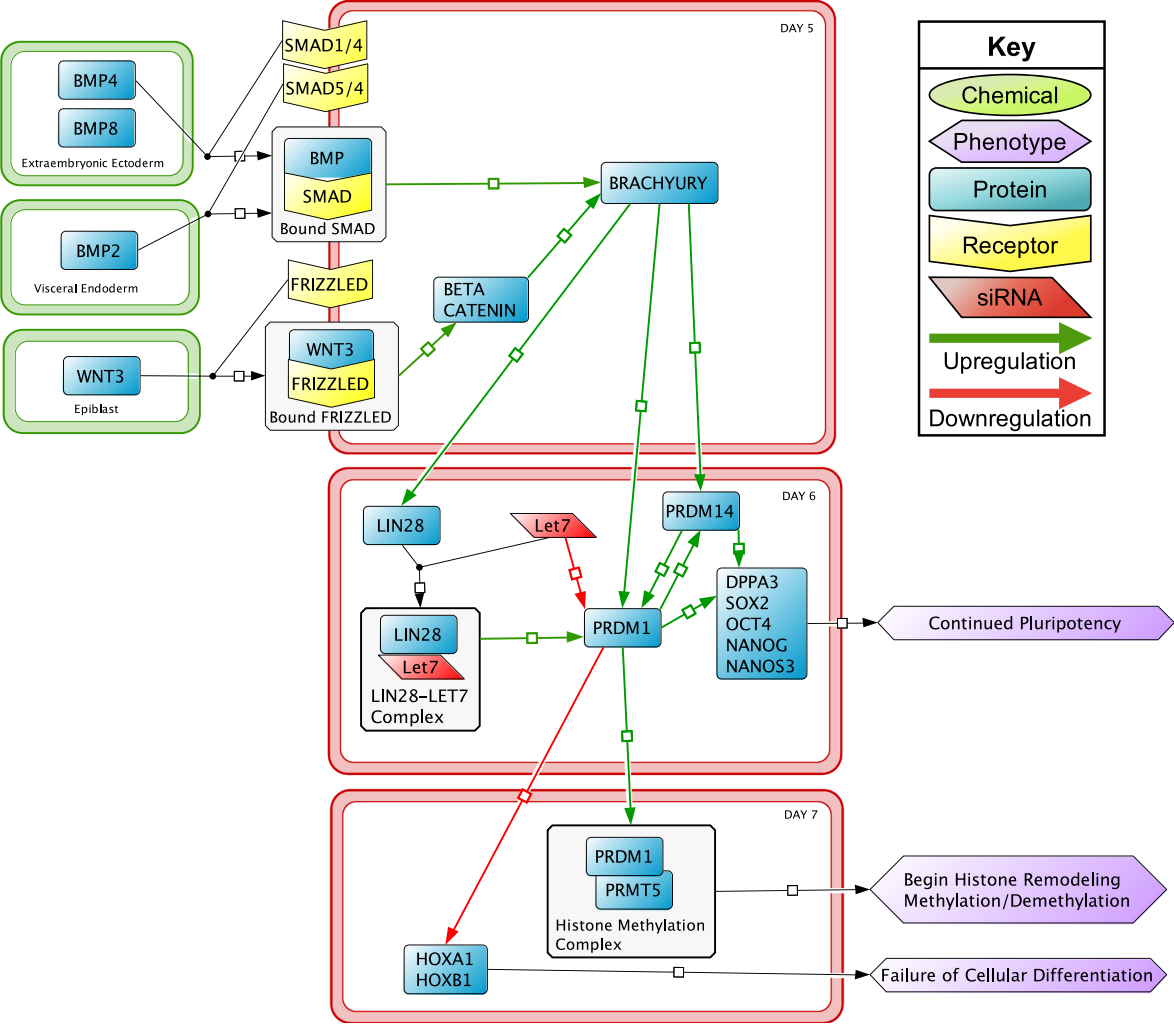
The initial signaling interactions in primordial germ cells begin on embryonic day 5 (E5) and are involved in the maintenance of pluripotency factors. Molecular events from E5 to E7 occur between the primordial germ cells (*red outline*) and embryonic tissues (*green outline*)

The initial appearance of six PGCs is identified by increased bone morphogenetic protein (BMP) and proto-oncogene wingless-type mouse mammary tumor virus integration site family member 3 (WNT3) levels (Fig. 2). BMPs (i.e., BMP2, BMP4, and BMP8) are extracellular signaling peptides that bind to mothers against decapentaplegic homologs 1 and 4 (SMAD1/4) and SMAD4/5 receptors [2, 16]. It is not known which BMP binds to which dimeric SMAD receptor. Studies have shown BMP4 alone is sufficient for PGC development, however BMP2 and 8 enhance the quantity of viable PGCs formed [17–19]. Activation of the Wnt3/β-catenin signaling pathway is also necessary to activate transcription factors that promote pluripotency and prevent differentiation [13]. WNT3 binds to the Frizzled-related receptor protein (FRIZZLED) on the surface of the PGC activating the β-catenin pathway, which leads to promotion of pluripotency factors through activation of Brachyury [20]. Binding of BMPs and WNT3 to their receptors also leads to the activation of Brachyury, an intracellular transcription factor.

From E6 to E7, Brachyury activates proteins that promote expression of pluripotency factors and histone methylation [20]. Histone methylation in the PGC helps maintain pluripotency by allowing transcription factors more access to regions of DNA. Brachyury activates positive regulatory domain zinc finger protein 1 (PRDM1), a transcription repressor, which blocks transcription of differentiation factors homeobox A1 (HOXA1) and HOXB1 [21]. PRDM1 additionally promotes activity of nanos homolog 3 (NANOS3), which leads to the expression of genes (e.g. sex determining region Y-box 2 (*Sox2*), octamer-binding transcription factor 4 (*Oct4*), and homeobox transcription factor (*Nanog*)) involved in maintaining pluripotency [22]. PRDM1 can form a dimer with protein arginine n-methyltransferase 5 (PRMT5), a histone methyltransferase, to dimethylate the genome at the arginine 3 residue of histone 4 preventing gene silencing [23]. Freeing the genome is necessary for meiotic division to produce functional gametes. PRDM1 and PRDM14, also a transcription factor, are able to upregulate each other to stimulate PGC development. Similar to PRDM1, PRDM14 plays a role in cellular pluripotency and is also involved in epigenetic reprogramming. PRDM14 activates developmental pluripotency associated 3 (DPPA3), a pluripotency factor, and enhances expression of *Sox2*, *Oct4*, and *Nanog* [24]. DPPA3, SOX2, OCT4, NANOG, and NANOS3 are all involved in the continued pluripotency of PGCs [24–26]. Protein lin-28 homolog A (LIN28) plays a role in maintaining pluripotency by forming a complex with a small inhibitory mRNA (siRNA), lethal 7 (LET7), to free *Prdm1* genes for transcription [27]. When LET7 is not bound to LIN28, LET7 binds to *Prdm1* inhibiting transcription and reducing pluripotency.

On E7, an increase in the levels of alkaline phosphatase (ALP) is observed in the Golgi apparatus of PGCs [28]. The dark staining properties of ALP allow PGCs to be identified in the embryo quite easily at this stage. It is unknown why ALP is expressed; however localization in the Golgi implies active protein synthesis. Studies of ALP knockout mice do not show inhibited PGC development or migration [29], which suggests that ALP is not critical for the process of ovarian development, but is important for identifying PGCs at this stage.

### PGC migration to the gonadal ridge (E8 to E9)

**Fig. 3:**
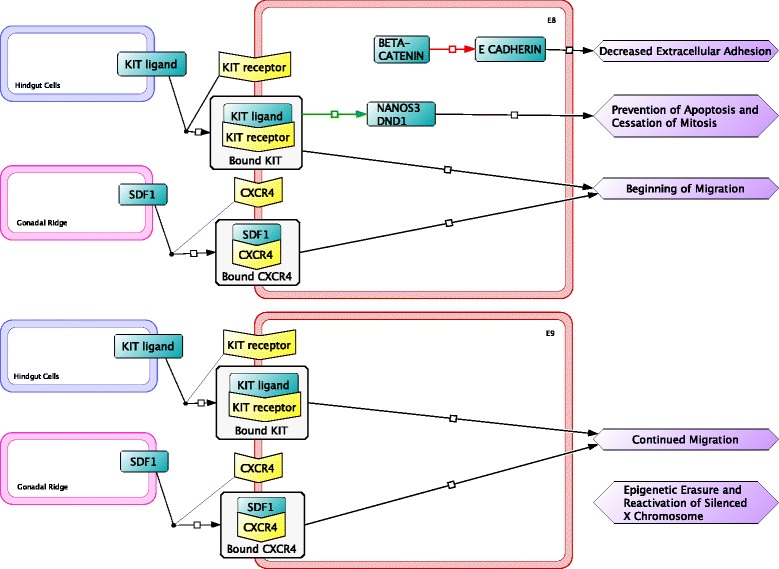
During embryonic day 8 (E8) to E9, adhesion between primordial germ cells is downregulated and expression of factors initiating migration signals are upregulated. The gonadal ridge (*pink outline*) and hindgut (*blue outline*) secrete chemoattractant compounds to primordial germ cells (*red outline*) that influence migration

PGCs originate in extraembryonic tissue posterior to the location of the future gonad, thus cellular migration must occur for the PGCs to become a part of the gonad in both males and females. PGC migration begins a couple days after the expression of pluripotency factors and initiation of histone methylation. Two schools of thought exist with respect to mitosis during PGC migration to the gonadal ridge: (i) before migration occurs, the founding population of PGCs must receive proper signals to halt mitosis and initiate factors necessary for cellular motility and migration [30]; and (ii) PGCs continue to proliferate during PGC migration [31, 32]. On E7.5 a population of PGCs is formed in the posterior region of the embryo awaiting migration signals. β-catenin production reduces E-Cadherin, decreasing PGC to PGC cellular adhesion (Fig. 3) [33, 34]. The decrease in adhesion allows the PGCs to become more motile and enhances their ability to migrate. PGCs have been observed to adopt polarized morphology with cytoplasmic protrusions just prior to migration, yet the factors causing these morphological changes is still unknown.

PGC migration begins on E8 and is mainly controlled through two separate ligand-receptor chemoattractant signaling interactions. Ligand proteins involved in PGC migration are secreted from the gonadal ridge and from the hindgut. The gonadal ridge is the region of tissue that will become the future gonad and the hindgut will develop into part of the gastrointestinal tract. The gonadal ridge secretes a ligand, stromal cell-derived factor 1 (SDF1), that binds to the chemokine receptor type 4 (CXCR4) receptor on the PGC causing the PGCs to migrate to the gonadal ridge [31, 35]. SDF1-CXCR4 mediated migration of PGCs was first observed in zebrafish embryos and later confirmed in mice [36, 37].

**Fig. 4:**
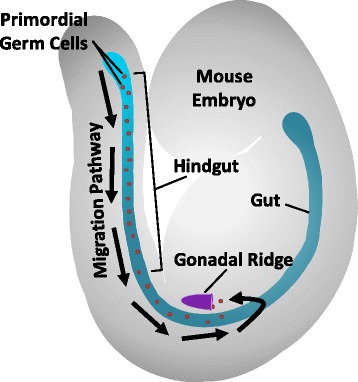
Migration of primordial germ cells (*red*) along the hindgut (*blue*) toward the gonadal ridge (*purple*) in the mouse embryo (*gray*) on embryonic day nine (E9). Arrows show the pathway of primordial germ cell migration

PGCs do not migrate in a direct path to the gonadal ridge, but rather move along the basal surface of the hindgut until they are parallel with the gonadal ridge and then migrate to the gonadal ridge (Fig. 4) [38]. The hindgut secretes the stem cell growth factor Kit (KIT) ligand (also called Steel factor) that binds to the KIT receptor (also called c-Kit) on the PGC. KIT ligand-receptor signaling plays a role in the motility of PGCs during migration as opposed to dictating the direction of migration [39]. Activation of the KIT signaling pathway also activates a tyrosine kinase and promotes survival factors like NANOS3 and dead end protein homolog 1 (DND1), that prevent apoptosis and halt mitosis of PGCs during migration [40].

**Fig. 5:**
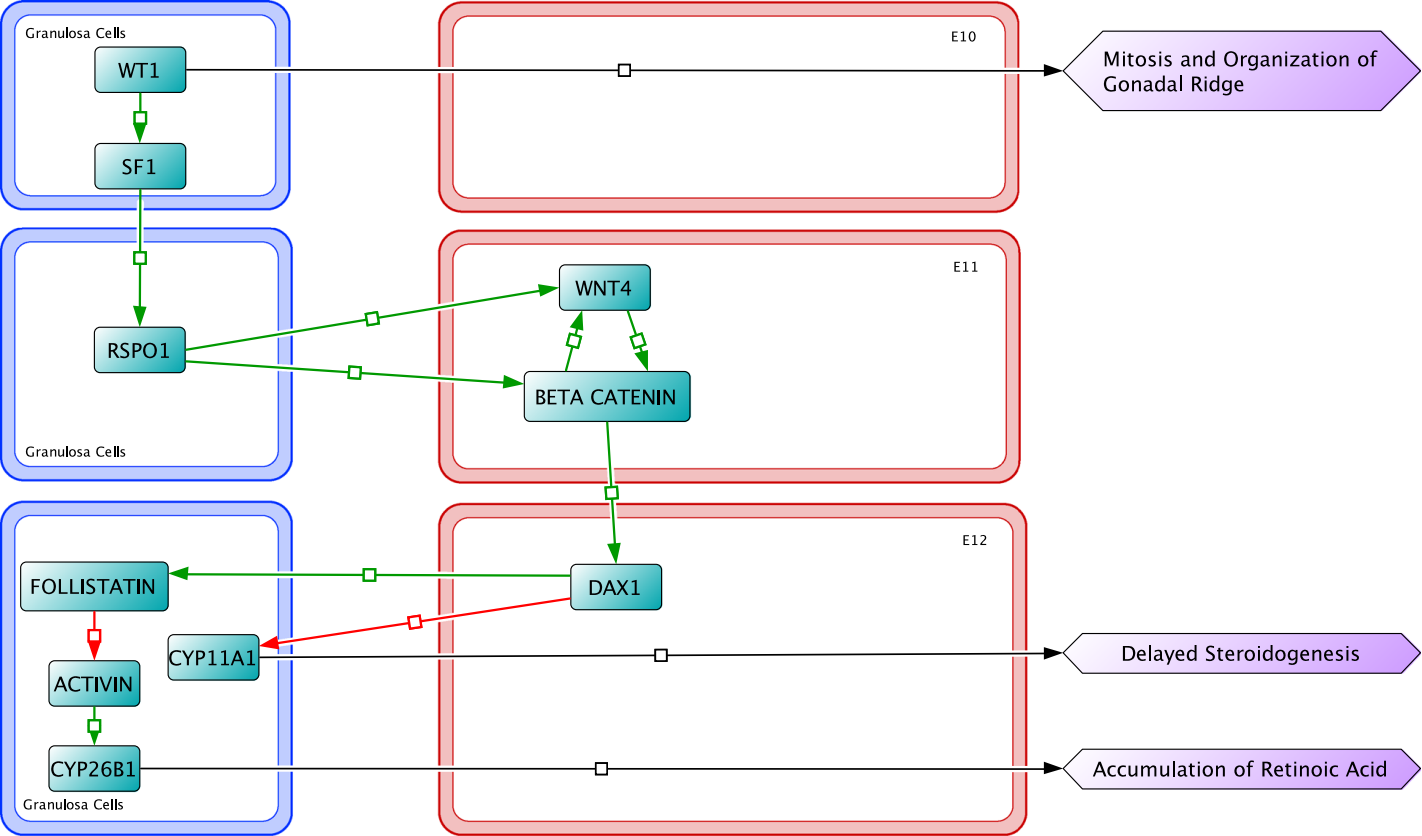
Signaling important in female sex differentiation, between somatic (i.e. granulosa) cells (*blue outline*) and oogonia (*red outline*), begins embryonic day 10 (E10) to E12

Survival factors are necessary to ensure that a sizable population of PGCs reach the gonadal ridge. It is proposed that survival factors are also involved in regulating apoptosis of cells that deviate from the migratory pathway. PGCs that end up in a region of the embryo too far from secreted survival factors will be signaled for apoptosis [2]. Factors involved in the survival of PGCs during migration include integrin beta-1 (ITGB1), 3-hydroxy-3-methylglutaryl-CoA reductase (HMGCR), DND1, and B-cell lymphoma 2 (BCL2)-associated protein X (BAX) [41]. ITGB1, HMGCR, and DND1 support survival of PGCs during migration, although are not critical to the process and were not included in the visual representation. BAX is an apoptosis regulator and also plays an important role in controlling the oocyte population later in early ovarian development [42]. On E10 (Fig. 5), the cessation of migration begins once the PGCs interact with somatic cells upon reaching the gonadal ridge.

### The bipotential gonad and sex differentiation (E10 to E15)

During PGC migration, cells in the gonadal ridge express Wilms tumor protein homolog (WT1) (Fig. 5), which promotes the thickening, organization, and growth of the gonadal ridge [43]. WT1 is additionally involved in PGC proliferation and female differentiation during this period [44]. In males and females empty spiracles homeobox 2 (EMX2) plays a role in development of gonads and other reproductive organs [45]. Another factor involved in gonad development is secreted during PGC migration, LIM/homeobox protein 9 (LHX9), which regulates somatic cell proliferation [46]. EMX2 and LHX9 are not included since neither factor is directly involved in the development of viable oocytes. Upon colonizing the undifferentiated gonadal ridge, PGCs undergo a period of rapid mitosis, dividing to 3,000 cells by E12.5 [47]. On E14.5 there are approximately 18,000 cells in the ovary and PGCs have developed to oogonia.

**Fig. 6:**
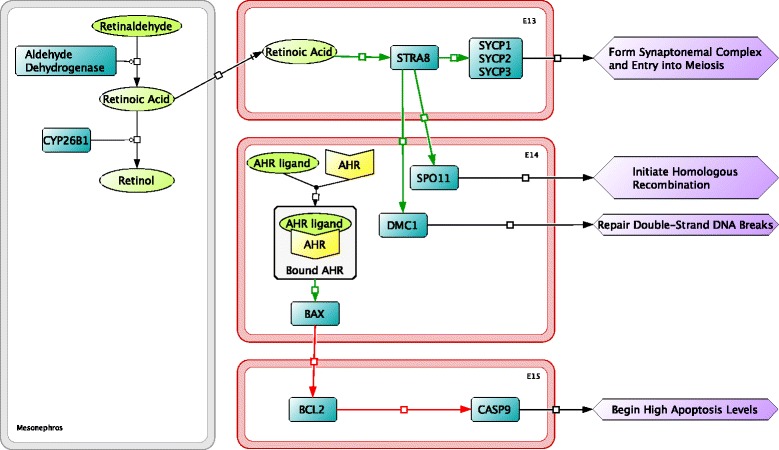
From embryonic day 13 (E13) to E15, molecular interactions involved in female sex differentiation in oocytes (*red outline*) and mesonephric cells (*gray outline*) continue. During this time period apoptosis of oocytes is initiated through activation of the aryl hydrocarbon receptor

Shortly after the period of mitosis, differentiation factors begin to be promoted. The normal development of the bipotenital gonad produces female reproductive organs, unless male-specific factors are upregulated; this critical juncture in the bipotential gonad is mediated by steroidogenic factor 1 (SF1) [2, 48]. Ovary formation occurs through uninhibited expression of female differentiation factors (R-spondin-1 (RSPO1), WNT4, β-catenin, and dosage sensitive sex reversal, adrenal hypoplasia critical region, on chromosome X, gene 1 (DAX1)), and the downregulation of cytochrome P450, family 11, member A1 (CYP11A1) (Fig. 5). RSPO1 plays an essential role in ovary formation by activating the WNT4/β-catenin signaling pathway on E11 [48, 49]. Upregulation of WNT4/β-catenin inhibits production of CYP11A1 delaying steroidogenesis and promotes production of female differentiation factors, FOLLISTATIN and DAX1 [50]. DAX1 plays a role in steroidogenesis inhibition and activation of steroidogenesis stimulated by retinoic acid 8 (STRA8) (see Fig. 6) [51, 52]. Male-specific factors were omitted because our focus is upon ovarian development. However, male sex differentiation would take place during this period with the expression of male-specific factors as described briefly below.

On E12, β-catenin controls follistatin signaling leading to the accumulation of retinoic acid through the downregulation of the retinoic acid metabolizing protein, CYP26B1 [53]. Follistatin binds to activin, regulating CYP26B1 (Fig. 5). CYP26B1 is a sex determination regulator involved in the degradation of retinoic acid. In males, CYP26B1 is upregulated which causes the breakdown of retinoic acid, suppressing *Stra8*-dependent meiotic pathway and a *Stra8*-independent mitotic pathway and promoting male germ cell differentiation [54].

The build up of retinoic acid stimulates STRA8 to activate factors that promote meiosis of oogonia during early ovarian development. STRA8 (Fig. 6) first activates synaptonemal complex proteins 1, 2, and 3 (SYCP1, SYCP2, and SYCP3), which play a role in the formation of the synaptonemal complex [55]. During meiosis the synaptonemal complex organizes and pairs homologous chromosomes for genetic recombination. STRA8 also activates meiotic recombination protein SPO11, and DNA meiotic recombinase 1 (DMC1), which are involved in initiating homologous recombination through double-stranded DNA breaks and repairing double-stranded breaks, respectively [56]. Double-stranded DNA breaks are necessary for recombination during meiosis. Oogonia develop into oocytes prior to entering meiosis.

After the period of mitosis and sex determination, some oocytes undergo apoptosis. Death of oocytes begins on E14.5 (Fig. 6) and continues until the end of early ovarian development. The number of oocytes decreases to 13,000 oocytes on E18 and to about 8,000 on P2 [47]. The apoptosis pathway is initiated through activation of the aryl hydrocarbon receptor (AHR). AHR activates BAX, causing BAX to bind with apoptosis regulator BCL2 allowing formation of an apoptosome in the cytosol of the oocyte [42]. Apoptosome formation activates caspase-9 (CASP9) leading to apoptosis [57]. It is proposed that the apoptotic process is activated to eliminate abnormal or inferior cells that may not produce healthy, fertilizable eggs during the later phase of ovarian development.

### Germ cell nest formation (E16 to E18)

**Fig. 7:**
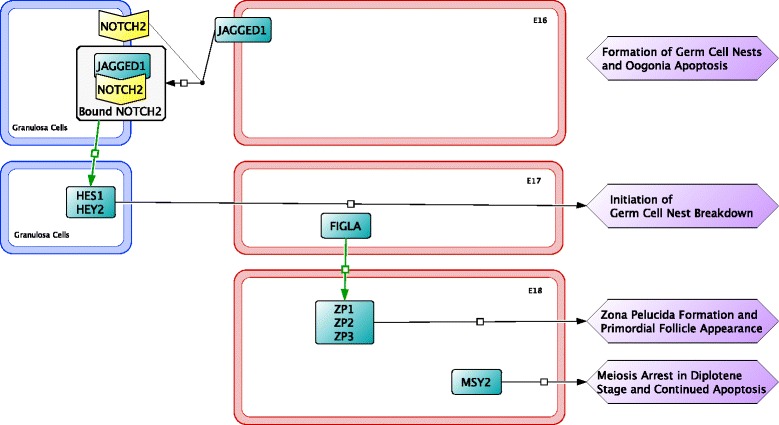
Signaling between granulosa cells (*blue outline*) and oocytes (*red outline*) initiating the formation of germ cell nests occurs from embryonic day 16 (E16) to E18. The exact signaling pathways for the formation of germ cell nests is unknown

During the period of oocyte apoptosis, oocytes form clusters called germ cell nests. Germ cell nests are interconnected oogonia formed by incomplete cytokinesis and surrounded by somatic cells, like granulosa cells [58]. Germ cell nest size and abundance, prior to birth, show no significant difference between cortex and medullar regions [47]. The topic of germ cell nest formation is not well studied, thus signaling factors involved in formation and the purpose of nest formation is unknown (Fig. 7).

### Germ cell nest breakdown and primordial follicle formation (E16 to P2)

Signaling factors involved in the breakdown of germ cell nests are expressed a few days prior to the actual breakdown of germ cell nests. On E16, the neurogenic locus notch homolog protein 2 (NOTCH2) signaling pathway is activated in granulosa cells by surface protein jagged-1 (JAGGED1) binding to the NOTCH2 receptor and continues into early postnatal stages [59, 60]. Activation of the NOTCH2 signaling pathway upregulates transcription factors hairy and enhancer of split-1 (HES1) and hairy/enhancer-of-split related with YRPW motif protein 2 (HEY2), which are necessary for germ cell nest breakdown and primordial follicle formation [61]. Factor in germline alpha (FIGLA), a transcription factor, is involved in the upregulation of zona pellucida sperm-binding protein 1 (ZP1), ZP2, and ZP3; proteins that are involved in recognition and binding of an oocyte to granulosa cells [62].

**Fig. 8:**
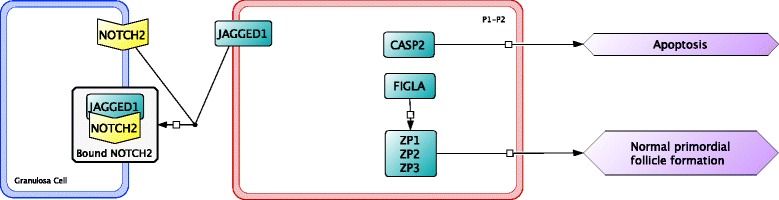
The last stage of early ovarian development occurs after birth from postnatal day 0 (P0) to P2 involving the continued breakdown of germ cell nests and formation of primordial follicles in granulosa cells (*blue outline*) and oocytes (*red outline*). The mechanism for these processes is also not well known

The final stage of early ovarian development occurs two days after birth with the breakdown of germ cell nests and formation of primordial follicles (Fig. 8). For the majority of mouse strains, the embryonic period ends and mice are born at about E20-21 (P0); at this stage, germ cell nests are present in the developing ovary. Breakdown of the germ cell nests is initiated after birth and is associated with a decrease in estradiol concentrations from the removal of the placental connection [43]. During the breakdown of germ cell nests, granulosa cells begin to invade and encircle a single oocyte, eventually forming a primordial follicle [63]. Primordial follicles are morphologically distinguished as a single oocyte, larger in size than one in a germ cell nest, completely surrounded by a single layer of flattened, squamous shaped granulosa cells. Oocytes in primordial follicles have a larger cytoplasm to nucleus ratio compared with oocytes in germ cell nests [64]. Primordial follicles formed during early ovarian development make up the pool of potential fertilizable eggs at sexual maturity. Follicles remain quiescent until activated through folliculogenesis. Those activated prior to puberty will undergo atresia during folliculogenesis, while those activated post-puberty may produce fertilizable eggs or also undergo atresia [65]. As with germ cell nest formation, the mechanisms of germ cell nest breakdown, and primordial follicle formation in mice are also not fully understood.

### Evidence for ovarian stem cells

In mammals, the abundance of potentially fertilizable germ cells was originally thought to be limited to the number of primordial follicles formed during early ovarian development, however studies have observed the presence of stem cells in the mouse ovary beyond P2. OSCs were discovered in the ovarian surface epithelium and presence of cells in the ovary was detected weeks to months after birth [66–68]. Controversy remains whether or not the observed stem cells actively contribute to the germ cell pool. A recent study found germ cells in the ovary expressing factors related to the entry of meiosis and germ cell mitosis up to 4 months after birth [6]. In contrast, cell-lineage tracing studies show no evidence for presence of putative germ stem cells in juvenile and adult mice [9–11]. The presence of active germ stem cells producing viable germ cells would modify our understanding of the size of the female germ cell pool, the female reproductive lifespan, and select infertility issues. With respect to our review and visual representation, OSCs may influence the size of the ovarian follicle population beyond P2. Our visual representation of early ovarian development would continue to reference the stages and timeline presented here, because this process is important for producing the founding population of primordial follicles in the ovary. Additional research is required to fully understand the elusive role of the mammalian OSC.

## Conclusion

This review summarizes the molecular signaling and regulation associated with the current scientific understanding of early ovarian development in mice. It is the first visual representation, to our knowledge, that shows the known signaling networks vital to ovarian development, elucidating the pathways connected to specific processes. The figures show how different molecular events are involved in similar developmental activities. For example, PGC migration is influenced by both SDF1-CXCR4 and KIT ligand-receptor interactions, though the two chemoattractant interactions are not controlled by the same molecular signaling. In addition, this study visually highlights areas of ovarian development that lack data, such as germ cell nest formation and germ cell nest breakdown.

Applications of this review include serving as a foundation for cell-based computational model development, using it as an education resource for early ovarian development instruction, and predicting adverse reproductive outcomes due perturbations (e.g. exposure to reproductive toxins) at the molecular and cellular levels. Phenotypic abnormalities resulting from toxicant exposure can be linked to a molecular event(s) in the visual representation, highlighting a potential biological target(s) of the compound. Information of the connection between phenotypic outcomes and biological targets can ultimately be used for a predictive computational model that simulates changes in morphology and cell counts based upon perturbations in molecular-level targets. While ovarian development is relatively well understood in mice, more research is needed to fill data gaps in our understanding of the ovarian developmental process.

## Abbreviations

Following the mouse nomenclature, proteins are abbreviated in uppercase letters and gene abbreviations use italicized upper and lowercase letters.

AHR, aryl hydrocarbon receptor; ALP, alkaline phosphatase; BAX, B-cell lymphoma 2 - associated protein X; BCL2, B-cell lymphoma 2; BMP, bone morphogenetic protein; CASP9, caspase-9; c-Kit, KIT receptor; CXCR4, chemokine receptor type 4; CYP11A1, cytochrome P450, family 11, member A1; CYP26B1, Cytochrome P450, family 26, member B1; DAX1, adrenal hypoplasia critical region, on chromosome X, gene 1; DMC1, DNA meiotic recombinase 1; DNA, Deoxyribonucleic acid; DND1, dead end protein homolog 1; DPPA3, developmental pluripotency associated 3; E*, embryonic day *; EMX2, empty spiracles homeobox 2; FGSC, female germline stem cell; FIGLA, factor in germline alpha; FRIZZLED, frizzled-related receptor protein; HES1, hairy and enhancer of split-1; HEY2, hairy/enhancer-of-split related with YRPW motif protein 2; HMGCR, 3-hydroxy-3-methylglutaryl-CoA reductase; HOXA1/B1, homeobox A1 or B1; ITGB1, integrin beta-1; JAGGED1, cell surface protein that is a ligand for multiple NOTCH receptors; KIT, stem cell growth factor (Kit ligand or Steel factor); LET7, lethal 7; LHX9, LIM/homeobox protein 9; LIN28, lin-28 homolog A; *Nanog*, homeobox transcription factor; NANOS3, nanos homolog 3; NOTCH2, neurogenic locus notch homolog protein 2; *Oct4*, Octamer-binding transcription factor 4; OGSC, ovarian germ stem cell; OSC, ovarian stem cell; P*, postnatal day *; PGCs, primordial germ cells; PRDM1/14, positive regulatory domain zinc finger protein 1 or 14; PRMT5, arginine n-methyltransferase 5; RSPO1, R-spondin-1; SDF1, stromal cell-derived factor 1; SF1, steroidogenic factor 1; siRNA, small inhibitory ribonucleic acid; SMAD*, mothers against decapentaplegic homologs (* = 1, 4 and 5); *Sox2*, sex determining region Y-box 2; SPO11, meiotic recombination protein SPO11; STRA8, steroidogenesis stimulated by retinoic acid 8; SYCP*, synaptonemal complex proteins (* = 1, 2, or 3); VSEL, very small embryonic-like stem cell; WNT*, wingless-type mouse mammary tumor virus integration site family member (* = 3 or 4); WT1, Wilms tumor protein homolog; ZP*, zona pellucida sperm-binding proteins 1, 2, or 3.
